# The effect of structural changes on the low strain rate behaviour of the intervertebral disc

**DOI:** 10.1177/09544119241272915

**Published:** 2024-08-24

**Authors:** Samantha Hayward, Patrick S Keogh, Anthony W Miles, Sabina Gheduzzi

**Affiliations:** Department of Mechanical Engineering, University of Bath, Bath, UK

**Keywords:** Biomechanical testing/analysis, dynamics [biomechanics], joint biomechanics, spine biomechanics, intervertebral disc

## Abstract

The annuus fibrosus (AF) and nucleus pulposus (NP) of the intervertebral disc (IVD) work in conjunction to dissipate spinal loads. In this study we have isolated the contribution of the NP to the overall response of the disc and investigated the effect of extreme structural changes to the disc on the mechanical behaviour. Linear stiffness, overall load range, hysteresis area and total energy were used to evaluate the impact of these changes on the spine and surrounding structures. Six porcine lumbar isolated disc specimens were tested in 6 DOFs with a 400 N compressive axial preload at low strain rates in three conditions: intact (IN), after total nucleotomy (NN) and after the injection of bone cement into the nuclear void (SN). The latter two conditions, NN and SN, were chosen to emulate the effect of extreme changes to the NP on disc behaviour. When comparing with intact specimens, significant changes were noted primarily in axial compression-extension, mediolateral bending and flexion-extension. NN and SN cases demonstrated significant increases in linear stiffness, overall load range and total energy for mediolateral bending and flexion-extension compared to the intact (IN) state. SN also demonstrated a significant increase in total energy for axial compression-extension, and significant decreases in the elastic contribution to total energy in all axes except flexion-extension. These changes to total energy indicate that surrounding spinal structures would incur additional loading to produce the same motion in vivo after structural changes to the disc.

## Graphical abstract

**Graphical Abstract:**
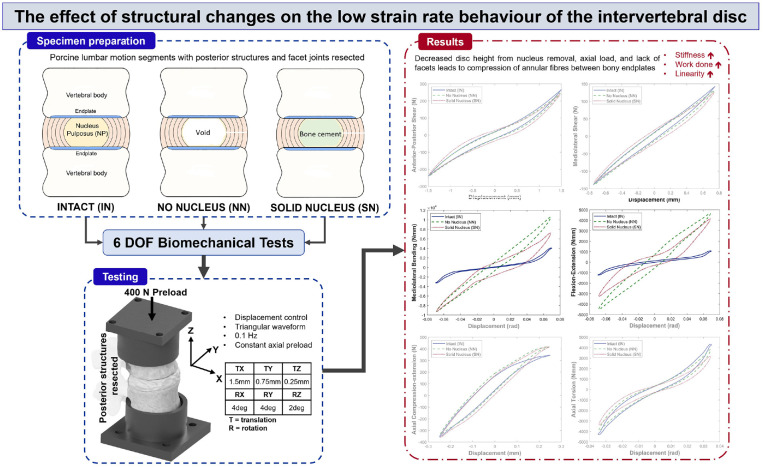

## Introduction

**Figure 1.**
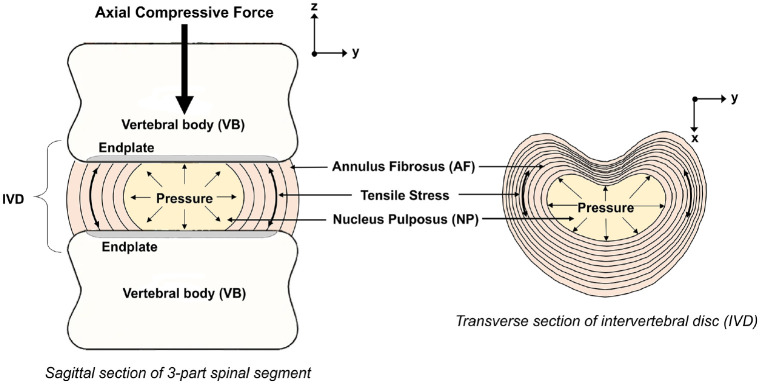
Illustration of the structures of the spinal symphysis.

The intervertebral discs (IVDs) act as flexible spacers between the rigid vertebrae of the spine, and function to absorb and dissipate loads and allow for relative motion between vertebrae.^1–3^ IVDs comprise two distinct regions: the outer annulus fibrosus (AF) which circumferentially encloses the inner nucleus pulposus (NP) (Figure 1). The annulus is able to withstand tensile strain in all directions and the gelatinous NP (Figure 1), when healthy, largely consists of proteoglycans which function to maintain the disc’s hydrostatic pressure.^4^

The mechanical behaviour of the IVD is influenced by the interaction between the annulus and the nucleus, and the flow of fluid to and from the IVD.^5^ A healthy disc in axial compression, undergoes bulging, resulting in tensile hoop stresses in the annular fibres (Figure 1).^2,6^ In a healthy disc, the two component structures work in tandem with the AF deforming under load, and the NP providing hydrostatic pressure, acting as a semi-compliant centre, facilitating dissipation of loads and rolling motion between the vertebrae.^7^ Disc degeneration causes biochemical and structural changes to the disc and these changes affect the mechanical response of the disc. Investigating how the component tissue structures of the disc contribute to the overall response is useful to further understanding of how degenerative changes affect the behaviour of the disc.

The biomechanical role of the NP has previously been investigated at high strain rates in axial compression.^6,8,9^ Other studies have investigated degrees of NP removal, replacement via discoplasty,^10–18^ and the effect of NP depressurisation at high strain rates.^6^ A number of studies investigate the removal and/or replacement of the NP in a more clinical sense with no biomechanical testing,^19,20^ while others perform biomechanical tests before and after induced changes, presenting data for 1–4 DOFs.^6,16,17,21,22^ There are also studies which grade cadaver discs with existing degenerative changes according to Thompson grading scale,^23,24^ with some performing 6 DOF biomechanical tests to assess the effects.^23^

Previous studies have investigated the effect of structural changes to the disc on the behaviour of functional spinal units (FSUs) with facet joints intact.^15,17,18,22,25–28^ The facet joints of the spine work in pairs to guide bending, twisting and extension, and limit shear and torsional motion. Therefore, maintaining the posterior elements in specimens means that changes to behaviour as a result of structural alterations to the disc cannot be isolated and evaluated.

There is a gap in the literature encompassing the effect of changes to the structural components of the intervertebral disc on the overall disc response. Previous studies have been limited by maintaining the facet joints in test specimens and therefore not isolating the disc response, and/or not performing biomechanical testing or in fewer than 6 DOFs.

Therefore, the aim of this study is to investigate the effect of imposed structural changes to the IVD on the low strain rate mechanical behaviour of the isolated IVD by performing biomechanical testing in all 6 DOFs.

In this study we have used isolated spinal discs (ISDs) with the facet joints resected. The removal of the facet joints allows the behaviour of the disc to be isolated. In this way we are able to infer what happens to the spinal symphysis and its surrounding structures as a result of changes to the structure of the disc and the native NP.

This study analyses the contribution of the AF and NP, to its mechanical response. This involved investigating the effect of extreme structural changes on the 6 DOF behaviour of the IVD. Linear stiffness, peak loads and elastic and dissipated energy were used as measures to evaluate the changes to disc behaviour after the induced structural changes.

## Materials and methods

Six lumbar spinal motion segments (2x L1L2, 2x L3L4 and 2x L5L6) were dissected from two fresh porcine spines. From these six, isolated spinal disc specimens (ISDs) consisting of the superior and inferior vertebral bodies, adjoining disc and anterior and posterior longitudinal ligaments were obtained. Following dissection, ISDs were sprayed liberally with 0.9% saline-solution, wrapped in saline-soaked tissue, triple-bagged and fresh-frozen at −20°C ± 2°C. For this preliminary study, porcine specimens were preferred to human specimens due increased control over lifestyle factors leading to reduced interspecimen variation. Of animal specimens commonly used in in vitro testing, including bovine, ovine and caprine, porcine anatomy is considered the closest to human which informed the choice made in this study.^29^

The behaviour of each ISD was investigated in its native state and following two extreme conditions of structural change performed sequentially. In the first case (denoted NN), the NP was completely removed from each specimen, simulating a central disc region with no hydrostatic pressure or resistance to deformation. In the second case (denoted SN), the void left by the removal of the NP was filled with a solid material, simulating a central disc region with no fluid characteristics and significant resistance to deformation.

To remove the nucleus (case NN), an incision was made through the right posterolateral annulus, central to the disc height, until no resistance was felt, indicating the nuclear capsule was breached. The incision was created with a type 10 scalpel blade. A set of curettes (details given in Supplemental Material and Supplemental Figure S1) was used to extract the nucleus by inserting the curette through the annular incision and using a combination of rotation and bending to traverse the full void. Specific effort was made to scrape the curette tool along the superior and inferior endplates and around the internal annular boundary to ensure as much nucleus material as possible was removed. Nucleotomy was assumed complete when three successive curettage motions yielded no additional material.

Extracted nucleus material was placed in a covered shallow glass petri dish half-filled with 0.9% saline solution to prevent the measured mass being confounded by lass of water content due to evaporation. The filled dish was weighed, before and after all the removed nucleus tissue was submerged, using a high precision digital scale (Mettler Toledo 220 g), the difference representing the mass of nucleus material. An estimate of loss of mass due to saline evaporation was obtained by repeating the experimental procedure over the maximum time taken to complete the nucleotomy (30 min) without placing any nucleus material in the dish. This yielded an estimate of a loss in mass due to evaporation of 0.063%. This represents the uncertainty in the NP mass measurements presented in this study. On average, 0.313 g (range: 0.18–0.42 g) of material was removed.

For case SN, the NP cavity was filled with bone cement (Palacos LV-30 Low Viscosity Cement with Gentamicin, Heraeus) using a 21-gauge regular bevel needle and syringe. Specimens were filled until a small amount of retrograde cement extrusion was noted, indicating complete filling. After curing, any retrogradely extruded cement was removed with forceps, ensuring no residue remained within the incision.

Both nucleotomy and cement filling procedures were performed on a dissection table with specimens held by hand and no preload applied.

**Figure 2.**
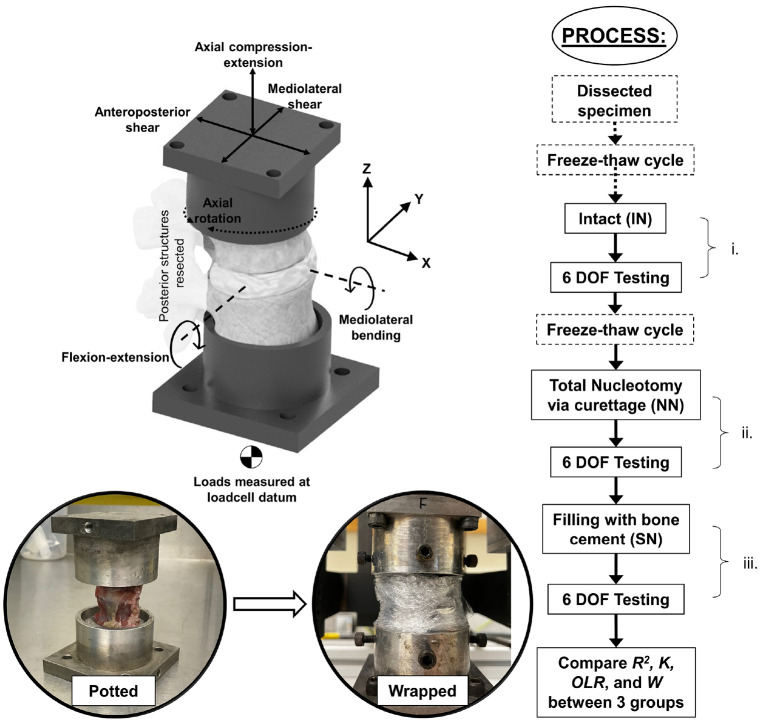
Illustration of specimen position, 6-axis coordinate axes used in tests, and process used during testing.

All testing was performed using a custom 6 DOF spine simulator^30^ operating under proportional-integral-derivative (PID) closed loop position control. Each of the six ISD specimens was tested in 6 DOFs with identical loading profiles in each of the three conditions (Figure 2) listed below:

- intact (IN),
- with no NP (NN),
- after cement filling to simulate a solid nucleus (SN).

Specimens underwent two freeze-thaw cycles which has been shown to have minimal effect on disc behaviour.^31,32^ Multiple freeze-thaw cycles have been shown to cause changes to the response of spinal specimens to load.^31,33^ Nevertheless, the use of fewer than three freeze-thaw cycles is not uncommon in studies of the spine.^15,28,32^

After dissection from fresh spines, specimens were frozen before testing in the intact state. Before testing, each specimen was fully thawed at a room temperature of (20°C ± 2°C) for 3.5 h. After IN tests, specimens underwent a second freeze-thaw cycle before NN and SN testing which was performed on the same day to remove the need for a third freeze-thaw cycle. Specimens were kept double-bagged while thawing to minimise moisture loss.^30^ Thawed specimens were then potted using Wood’s metal, ensuring the disc was parallel to the horizontal. Specimens were kept hydrated during testing according to the standard protocol whereby the exposed portion was sprayed with 0.9% saline solution, then wrapped in saline-soaked tissue and plastic wrapped to minimise dehydration.^34,35^

Before testing, specimens were pre-conditioned with a 400 N axial preload, simulating in vivo head and torso weight, ramped up over 15 min, then maintained for 30 min to equilibrate.^30^ Preload was maintained during testing. A constant velocity, triangular displacement waveform was applied at 0.1 Hz with amplitudes not exceeding lumbar range of motion (*ROM*) and similar to amplitudes used previously in the literature.^30,36^ The amplitudes, applied at the centre of the intervertebral disc, were ±1.5 mm in anterior-posterior shear, ±0.75 mm in mediolateral shear, ±0.25 mm in axial compression-extension, and ±4° in mediolateral bending and flexion-extension and ±2° in axial rotation.^36,37^ Each specimen underwent five displacement cycles in each axis. Testing was performed at room temperature (20°C ± 2°C). Output signals were acquired at 100 Hz; loads via a six-axis load cell (Figure 2) (AMTI MC3-A-1000; Advanced Mechanical Technology, Inc., MA, USA) and displacements via motor encoders (HEDL 5540, Maxon Motor UK, HSR35BCSS, THK UK, HFUC-17-80-2UH-SP+EC90+HEDL5540, Harmonic Drive UK).

The final three cycles in each axis were used to plot the load versus displacement curve for each of the six principal stiffness matrix elements, that is, where displacement and load are coaxial. According to the established method, stiffness ( K ) was estimated using linear least squares.^38^ Overall Load Range (*OLR*), an indication of peak loads, was calculated as the average of the difference between minimum and maximum loads during the final three cycles. An increased *OLR* whereby increased peak loads are seen for the same applied displacements indicates a decreased range of motion (*ROM*), similarly a decreased *OLR* indicates an increased *ROM*. Hysteresis area ( HA ), a measure of dissipated energy per cycle, was determined by quantifying average area enclosed by each loading cycle. Matlab R2020b (Mathworks Inc., Natick, MA, USA) was used for data analysis.

**Figure 3.**
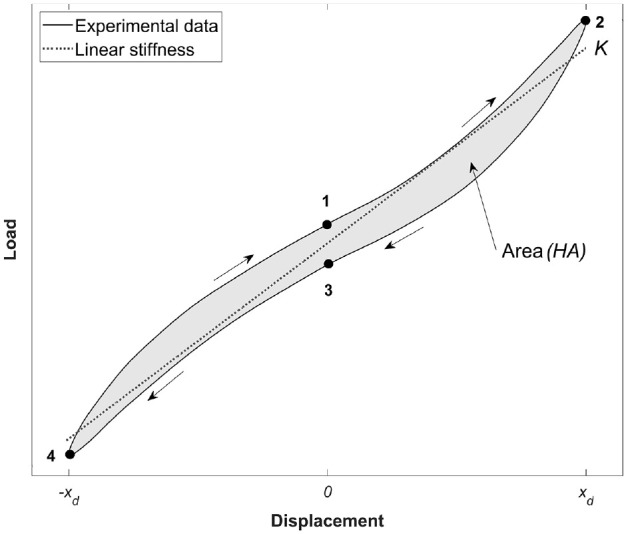
Illustration of the estimation of total energy as determined using the experimental load-displacement curve. The cycle can be divided into four regions, 1→2, 2→3, 3→4 and 4→1. Where, by convention, the cycle starts at 1.

The energy components per cycle are:

(1) $$W=\underset{\left(stored\;elastic\right)}{\underset{︸}{e}}+\underset{\left(dissipated\right)}{\underset{︸}{HA}}$$

where W , is the total energy, e is the stored elastic energy and HA is the hysteresis area enclosed by the load-displacement curve (Figure 3).

The stored elastic energy, *e *, in each cycle comprises four components:

(2) $$e=\frac{1}{2}K\left(\underset{1\to 2}{\underset{︸}{{x_{d}}^{2}}}+\underset{2\to 3}{\underset{︸}{{x_{d}}^{2}}}+\underset{3\to 4}{\underset{︸}{{\left(-x_{d}\right)}^{2}}}+\underset{4\to 1}{\underset{︸}{{\left(-x\right)}^{2}}}\right)$$

where K is the linear stiffness and $x_{d}$ is the displacement amplitude. The underbrace numbers 1–4 are denoted on the load-displacement cycle plot in Figure 3. By convention in the figure, the cycle is taken to start at position 1.

The total energy as defined here is equivalent to the work that would be done by muscular action in vivo overcoming the resistive force to motion, in both directions.

The contribution of the stored elastic energy, e , to the total energy, *W *, is denoted as $e_{e}$ :

(3) $$e_{e}=\frac{e}{W}$$

The samples were subject to sequential structural changes and measurements were repeated at each stage i, ii and iii (Figure 2). Statistical analysis was performed using IBM SPSS Statistics 28.0.0.0 (IBM Corporation, Armonk, NY, USA). Comparisons of data ( K , *OLR*, $e_{e}$ and W ) were made using one-way ANOVA with Bonferroni post-hoc tests. Significance of differences in mean values between (IN) and (NN), and (IN) and (SN) was assumed for *p* < 0.05.

## Results

**Figure 4.**
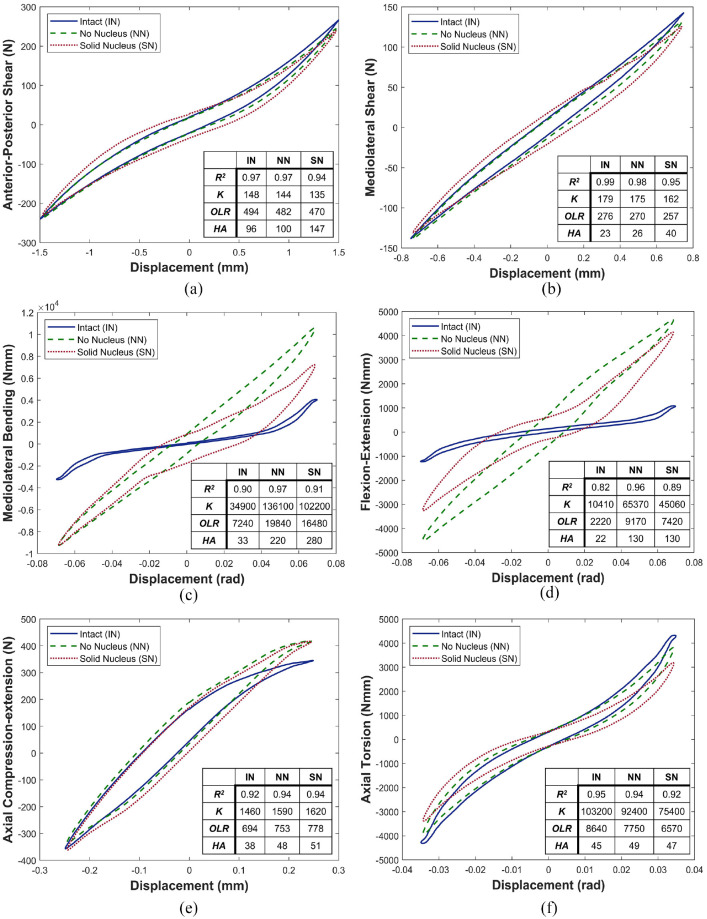
Average load-displacement curves of all specimens tested in (a) anterior-posterior shear, (b) mediolateral shear, (c) mediolateral bending, (d) flexion-extension, (e) axial compression-extension and (f) axial torsion. IN refers to intact, NN no nucleus and SN solid nucleus. Values are shown for *R*^2^, Stiffness, *K* (units: N/mm, Nmm/rad), Overall load Range, *OLR* (units: N, Nmm) and Hysteresis Area, *HA* (units: Nmm, Nmmrad). Area units are also equivalent to mJ.

Figure 4 shows the behaviour in the 6 DOFs and all three conditions for the average behaviour of all specimens. Both removal of the nucleus and its replacement with a solid material had limited impact on specimen behaviour in anterior-posterior shear and mediolateral shear (Figure 4(a) and (b), respectively), and in axial compression-extension and axial torsion (Figure 4(e) and (f), respectively). Changes were mainly confined to mediolateral bending and flexion-extension axes (Figure 4(c) and (d), respectively).

Figure 4(c) and (d) illustrate changes in the mediolateral bending and flexion-extension load-displacement curves, respectively, of average specimen behaviour between the three conditions: (IN), (NN) and (SN). In both axes, the linearity of behaviour is increased after nucleus removal, though some recovery of nonlinearity was noted in the solid nucleus (SN) specimens (Figure 4(c) and (d)).

**Figure 5.**
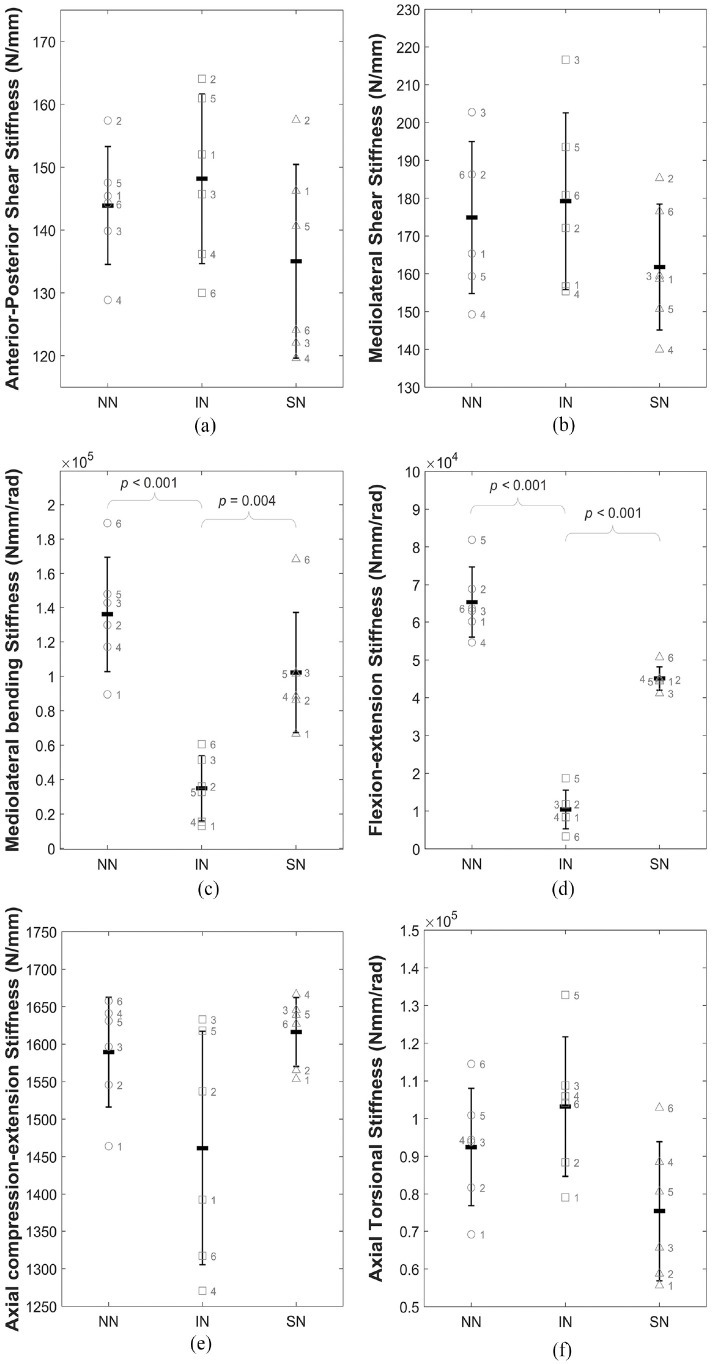
Illustration of the change in stiffness (*K*) for all specimens across the three conditions (intact (IN), no nucleus (NN) and solid nucleus (SN)) for (a) anterior-posterior shear, (b) mediolateral shear, (c) mediolateral bending, (d) flexion-extension, (e) axial compression-extension and (f) axial torsion. Data is presented as mean and standard deviation of the whole sample group with the individual labelled data points overlaid on the plot. Braces indicate statistically significant differences.

Figure 5 illustrates the stiffness of specimens between the three conditions: (IN), (NN) and (SN). Stiffness was significantly increased in NN and SN states in both mediolateral bending and flexion-extension (Figure 5(c) and (d)).

Table 1 presents the mean and range of the $R^{2}$ values, an output from the linear least squares fit. $R^{2}$ was used to give insight into the quality of linear fit to the experimental data for the six specimens. The linearity of the load-displacement curves changed more so in mediolateral bending and flexion-extension (shaded in Table 1). Complete removal of the nucleus (NN) caused an increase in linearity compared to intact (IN) specimens (8.60% and 17.4% in mediolateral bending and flexion-extension respectively). The introduction of a solid medium into the nuclear void resulted in recovery of nonlinearity in mediolateral bending, with the mean *R*^2^ of solid nucleus (SN) specimens equal to that of intact (IN) specimens. In flexion-extension, the mean *R*^2^ of the solid nucleus (SN) specimens was still greater (8.5% higher) than intact (IN) specimens, indicating more linear behaviour in specimens with a solid nucleus. In shear and torsion, there was little difference (<0.95%) between conditions (IN) and (NN), and changes <3.9% were noted between (IN) and (SN). In axial compression-extension, all changes were below 1.7%.

**Table 1.** Mean and (range) *R*^2^ values, a measure of the linearity of the load-displacement curves, calculated for the full load-displacement behaviour along the six principal axes for intact (IN), no nucleus (NN) and solid nucleus (SN) specimens.

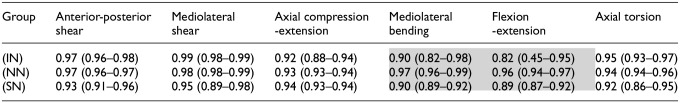

Shading indicates the greatest changes to linearity as seen in mediolateral bending and flexion-extension.

Table 2 presents the *p-*values for the mean stiffness and overall load range for the full six specimens in all three states. Specimens with no nucleus (NN) were significantly stiffer than intact (IN) specimens in mediolateral bending and flexion-extension with *p*-values <0.001 (Table 2). The introduction of a non-deformable solid nucleus (SN) also caused significant increases in mediolateral bending stiffness (*p*-value = 0.004) and flexion-extension stiffness (*p*-value < 0.001) compared to the intact (IN) condition (Table 2).

**Table 2.** *p*-values (*p* < 0.05) for stiffness ( K ) and overall load range (*OLR*) means between intact (IN) and no nucleus (NN) conditions, and intact (IN) and solid nucleus (SN) conditions.

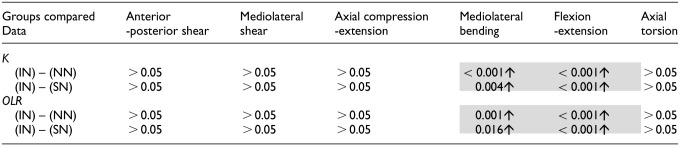

Increases and decreases compared to the intact (IN) state are indicated with an upwards or downwards pointing arrow, respectively. Non-significant differences (*p* > 0.05) are not shown. Shading indicates statistically significant changes to the data presented here: stiffness (K) and overall load range (OLR).

**Figure 6.**
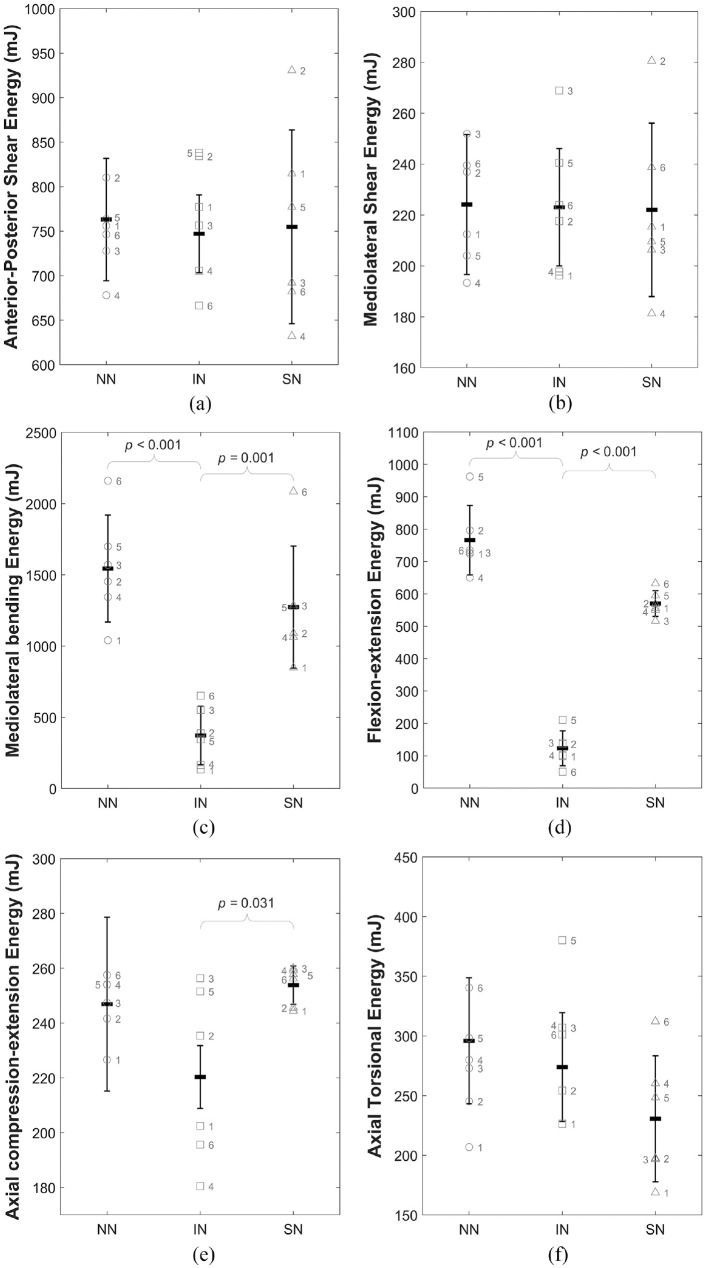
Illustration of the change in total mean energy (*W*) for all specimens across the three specimen conditions (intact (IN), no nucleus (NN) and solid nucleus (SN)) for (a) anterior-posterior shear, (b) mediolateral shear, (c) mediolateral bending, (d) flexion-extension, (e) axial compression-extension and (f) axial torsion. Data is presented as mean and standard deviation of the whole sample group with the individual labelled data points overlaid on the plot. Braces indicate statistically significant differences.

Figure 6 illustrates the total energy per cycle ( W ) for the three states IN, NN and SN for all 6 DOFs. Significant differences were noted only in axial compression-extension, mediolateral bending and flexion-extension. In these axes the intact (IN) condition represents the lowest energy condition, with both (NN) and (SN) states requiring significantly increased total energy per cycle.

Table 3 presents the *p-*values, demonstrating the significance of changes to the total energy per cycle ( W ) and the elastic contribution to the total energy ( $e_{e}$ ) for the full six specimens in both extreme conditions compared to the intact condition. Complete removal of the nucleus (NN) resulted in a significant increase in the total energy ( W ) in mediolateral bending and flexion-extension, as well as a decrease in the elastic contribution ( $e_{e}$ ) in mediolateral bending. The introduction of a solid material into the nuclear void (SN) significantly decreased the elastic contribution in all DOFs except flexion-extension, and significantly increased the total energy in axial compression-extension, mediolateral bending and flexion-extension (Table 3 and Figure 6).

**Table 3.** *p*-values (*p* < 0.05) for mean percentage elastic contributions ( $e_{e}$ ) to the total energy, and mean total energy ( W ) values between intact (IN) and no nucleus (NN) conditions, and intact (IN) and solid nucleus (SN) conditions.

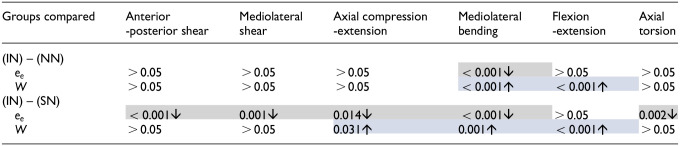

Increases and decreases compared to the intact (IN) state are indicated with an upwards or downwards pointing arrow, respectively. Non-significant differences (*p* > 0.05) are not shown. Grey and blue shading indicates statistically significant decreases and increases respectively in the data presented here: mean percentage elastic contributions (e_e) and mean total energy (W).

**Figure 7.**
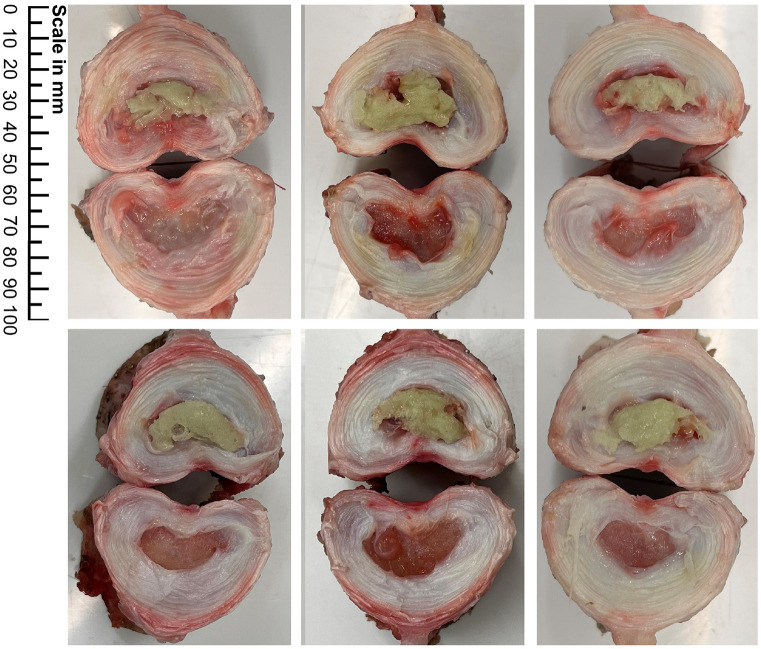
Bone cement distribution in the 6 specimens.

After testing, specimens were dissected and photographed to assess bone cement fill quality (Figure 7). Observations were made on completeness of filling and cement outside of the nucleus cavity. No cement was found to have extruded outside of the nucleus cavity. All specimens presented with good fill quality. Some small voids in the cement bolus were noted (Figure 7), likely caused by air bubbles or space occupied by remnants of nucleus tissue preventing cement from fully filling the NP cavity.

## Discussion

In axial torsion, $R^{2}$ is largely unchanged between stages (IN) and (NN), and (IN) and (SN) (Table 1). There are also no significant changes in K and *OLR* (Table 2 and Figure 4(f)). In shear, $R^{2}$ is largely unchanged between stages (IN) and (NN), and (IN) and (SN) (Table 1). Further, no significant changes in K and *OLR* were noted in these DOFs (Table 2 and Figure 4(a) and (b)). The annulus, which is responsible for bearing the majority of shear loads, was subject to minimal structural damage in the form of a posterolateral radial incision of width ≤7.2mm , the width of the scalpel blade used. Results demonstrate that both extreme changes to the nucleus have no significant effect on stiffness or *OLR* of the disc in response to shear. This indicates that changes to the hydrostatic pressure of the native, intact NP have no appreciable effect on the behaviour of the IVD in shear, and that the AF plays a more crucial role in load transfer in these axes (anterior-posterior shear, mediolateral shear and axial torsion). Due to the removal of the facet joints in our specimens, pure shear was possible. However, shear is not a natural in vivo motion, and while being potentially influential in spinal injuries,^39^ typically arises in conjunction with bending due to motion coupling.^38,40^

There is no significant change to axial compression-extension linearity ( $R^{2}$ ) or stiffness ( K ) after complete removal of the native nucleus (Tables 1 and 2 and Figures 4(e) and 5(e)). With small displacements applied during testing in the presence of an axial preload, which causes radial bulging of the annulus, the compressed annulus acts as a flexible spacer in lieu of the NP and therefore no significant changes to stiffness are evident. Direct axial compression has been shown to induce circumferential hoop tension in the fibres of the annulus, a state in which the annulus is uniquely strong. In vivo, the lumbar spine is constantly subjected to an axial load from head and torso weight, and the method of replicating this in vitro using an axial compressive preload is standard in the field.^41–43^

Replacing the native nucleus with a non-deformable solid material, bone cement, resulted in no significant differences in axial stiffness or linearity of behaviour (Tables 1 and 2 and Figures 4(e) and 5(e)). Bone cement, while a significantly stiffer material than the native nucleus^44^ and lacking its semi-fluid characteristics, does not reinstate the hydrostatic pressure provided by the native nucleus. Under compression, this hydrostatic pressure provides substantial resistance to load. When the nucleus is replaced by a non-deformable solid with no fluid characteristics, this interaction is no longer possible. Standard bone cements similar to that used in this study have been shown to have elastic moduli greater than three times those of vertebral bone.^44^ It is likely that subject to the 400 N axial preload and subsequent further axial displacements, the substantially higher modulus of the cement region caused deformation of the endplates and vertebral bone, allowing compressive displacement with load increases similar to the intact state.

In mediolateral, side-to-side, bending, and flexion-extension, forwards and backwards bending, in conjunction with the maintained axial preload, complete removal of the nucleus caused significantly increased stiffness, peak loads and total energy (Tables 2 and 3 and Figures 4(c), 4(d), 5(c) and 5(d)). This indicates that the hydrostatic pressure provided by the intact NP is crucial to maintaining normal load levels, stiffness values and energy expenditure in bending. Figure 4(c) and (d) illustrate this effect for a representative specimen and, alongside Figure 5(c) and (d), clearly show the significant increase in stiffness of the load-displacement behaviour of the disc. The significant increase in overall load range compared to the intact specimens indicates a reduced range of motion as greater loads are incurred for the same applied displacements.

Complete removal of the native nucleus resulted in significantly increased total energy in mediolateral bending and flexion-extension (Table 3), indicating that more energy is dissipated within the system compared to the intact (IN) state. This demonstrates that in these DOFs, the native nucleus is crucial to minimising the total work done by surrounding spinal structures. Cannella et al. similarly found that removal of the native NP resulted in increased hysteresis area (representative of dissipated energy) in mediolateral bending, flexion-extension and axial rotation.^16^

Many studies have demonstrated a decrease in stiffness following nucleotomy in FSUs and ISDs.^16,18,25,45–48^ Of the afore-mentioned studies,^18,25,45,46^ apply no axial preload during tests and,^16,47,48^ apply very small axial preloads, an order of magnitude lower than that applied in this study. Cannella et al. reported decreased stiffness in lower levels of mediolateral bending and flexion-extension in human ISDs tested after nucleotomy.^16^ However, they showed that at higher levels of moments the stiffnesses became equal to or greater than the intact condition.^16^ Techens et al.^15^ reported no significant changes in flexion, extension or lateral bending of their porcine FSUs after either nucleotomy or replacement of the nucleus by bone cement. However, while not statistically significant, their results showed an increase in specimen flexion and extension stiffness after nucleotomy and an increase in extension stiffness after the introduction of bone cement.^15^ Huang et al.^22^ showed a reduced ROM in mediolateral bending and a reduced neutral zone, indicating greater linearity of behaviour after the introduction of bone cement into their porcine FSUs.

The results from this study have shown significant increases in mediolateral bending and flexion-extension stiffness and mean energy of porcine ISD specimens after nucleotomy and NP replacement with bone cement. In this study, specimens were subjected to a 400N axial compressive preload that was maintained during testing to represent the in vivo condition.^30^ Many studies do not apply a preload and/or perform only preconditioning of samples.^15,18,26^ Others apply only a small axial compressive preload, much reduced compared to that applied in this.^6,8,9,16,28^ Removal of the native nucleus has been shown to decrease disc height and removal of the facet joints further decreases the spacing between vertebrae. Therefore, it is likely that under the preload applied in this study, the compression of the remaining annulus was such that the applied mediolateral and flexion-extension rotations were resisted by the annulus fibres compressed between the vertebral endplates, giving rise to higher loads for the same displacements and therefore, significantly higher stiffnesses. This greater resistance to motion is likely also responsible for the increased energy expended during motion. A study by Yang et al.^49^ presents a model of a human lumbar ISD which demonstrates increased stiffness in mediolateral bending and flexion-extension after removal of the nucleus, when the bending motions were combined with compressive loading similar to the axial preload applied in this study. Isolated bending motions with no compressive load resulted in decreased stiffness after nucleotomy.^49^

In both mediolateral bending and flexion-extension, replacing the native nucleus with a solid material resulted in significantly increased stiffness and peak loads, indicating reduced *ROM* (Table 2). The bone cement was injected into the nuclear void of specimens outside of the testing apparatus. However, at the point of cement injection, specimens were already potted in Wood’s metal for fixation in the testing apparatus. Therefore, it is likely, due to the weight of the superior pot and Wood’s metal mass, the injected cement filled only the void left by the concavity of the endplates and did not act to restore disc height. This, in conjunction with the applied compressive preload could have led to a similar effect as in the nucleotomy tests, with bending loads resisted purely by compression of the annular fibres between the outer endplate rims. This could explain the notably higher loads in these axes. Techens et al.^15^ reported no significant changes to *ROM* or stiffness in either DOF after a simulated discoplasty using bone cement in porcine lumbar FSUs. They assessed the biomechanical behaviour of lumbar spine specimens before and after replacing the native nucleus with bone cement. The current study focusses on the behaviour of the isolated intervertebral disc whereas^15^ maintained the posterior elements and facet joints, studying FSUs with no axial compressive preload maintained during testing. The notable load-sharing contribution and vertebral spacing of the facet joints, specifically in bending, impedes direct comparisons between the findings reported here and those of Techens et al.^15^

By working in pairs the facets prevent excessive bending, hyperextension and hyperflexion by limiting the ROMs.^50–52^ The facet joints are responsible for bearing varying levels of loads during spinal motions and studies have shown that the facets bear 10%–20% of the compressive load on the spine while in a neutral position, and approximately 50% of the anterior shear load during forward flexion.^50^ Changes to the disc following structural disruptions or degenerative changes can increase the load-sharing in the facets significantly.^3^ Ivicsics et al.^27^ showed that the portion of load supported by the facet joints in porcine lumbar specimens was significantly increased following nucleotomy over their range of flexion-extension values. The results from this study indicate that severe structural changes to the nucleus significantly affect the integrity of the disc and its ability to maintain vertebral spacing. Further, these results demonstrate the importance of the facet joints in maintaining vertebral spacing after structural changes and damage to the disc, preventing possible damage to the endplates and vertebral bone. A measurement of disc height at all stages in this study would have enabled a more thorough discussion of this topic.

The behaviour of the annulus is expected to be nominally symmetric, therefore, the increased behavioural symmetry after nucleotomy of the ISD specimen is an indication that the annulus is supporting the majority of loads (Figure 4(c) and (d)). Some asymmetry was regained after the inclusion of a solid medium in the nuclear void. The posterior positioning of the nucleus with respect to the geometric centre of the disc is a likely contributor to this: the cement bolus acts as a bearing allowing the VBs to roll with respect to one another, replicating to a certain degree the function of the native nucleus. However, total energy was also significantly increased, indicating that more energy is required to reach the same position. In vivo, this represents additional work that would be done through muscle activation and ligament action and these structures would therefore be subject to higher loads and greater strain in response to a stiffening of the nucleus.

The elastic contribution to the total work done ( $e_{e}$ ) was significantly decreased in all 6 DOFs except flexion-extension following introduction of a solid nucleus (SN) (Table 3), indicating that a stiffer nucleus requires significantly more dissipated energy to achieve the same *ROM*. With a solid cement bolus in place of the native nucleus, there is no hydrostatic pressure in the disc and the natural interaction with the annulus is disrupted, forcing the annulus to support more load. This is also supported by the data in Table 3 which shows a significant increase in total energy ( W ) in axial compression-extension, mediolateral bending and flexion-extension following the introduction of a solid nucleus.

As evident in Figure 6, the native (intact (IN)) condition represents the lowest total energy state, with both extremes causing significant increases in total energy required to reach the same displacement. This effect has been isolated to the IVD in this study, with the work done by the motors of the testing apparatus in overcoming specimen resistance to motion. However, in vivo it is the surrounding spinal structures like the facet joints, ligaments and musculature which act to generate displacement against resistance to motion. During this process, the muscles act to overcome forces resisting motion. An increase to total energy demonstrates an increased resistance to this motion and from this it can be inferred that in vivo, the surrounding spinal structures would have to act against greater resistive forces when the state of the NP approaches either of these extremes, in order to achieve the same displacements seen in the intact (IN) state.

In this study, six specimens were tested in three different states, IN, NN and SN. Repeat testing and excessive preconditioning can impact the measured response of spine specimens in in vitro testing due to factors such as dehydration of tissues or damage due to large movements. While a control group would have reduced these concerns, care was taken in the study to ensure applied motions were not in excess of normal porcine lumbar ROMs, aligning applied displacements with the literature. Furthermore, specimens were kept hydrated throughout all stages of preparation and testing, and applied preload was released between testing stages to allow specimens to recover and to prevent excessive preconditioning.

This study aimed to assess the effect of structural changes to the components of the isolated IVD. Since the nature of this study is preliminary, the use of porcine spines rather than human cadaver spines was preferred. Porcine spines are frequently used in in vitro studies^15,29,30,53^ and are preferred for the lower inter-specimen variability in anatomy, degeneration and biomechanical properties due to the control over animals’ lifestyle, nutrition and age. While the results from in vitro testing on porcine spines cannot be directly translated to humans, the trends in results can inform human studies.

In this study, an average of 0.313 g (wet weight) of material was removed during nucleotomy, in line with the range seen in the literature. Similar studies report the removal of approximately 1 g of nucleus material in cadaver discs,^27^ and 0.2 g in ovine and bovine discs.^46,48^ Dry weight is often used as an alternative metric to prevent the effect of hydration and water content affecting the results. Figure 7 shows each of the specimens after dissection through the midplane of the disc. As evident in this figure, the degree of nucleus removal was high, with little to no material remaining.

## Conclusions

The purpose of this study was to evaluate the contribution of the NP to disc behaviour in response to 6 DOF cyclic motion by inducing extreme structural changes and investigating the effects on behaviour, compared to the native disc. It was found that both removing and replacing the native NP significantly affects the stiffness, peak loads and total energy during applied motion at 0.1 Hz, specifically in mediolateral bending and flexion-extension. This demonstrates that the hydrostatic pressure provided by the native NP is critical in maintaining normal levels of stiffness, peak loading, energy dissipation and total energy at low strain rate cyclic motion, particularly in bending. The findings from this study give insight into the interaction between the annulus and the nucleus of the IVD, and biomechanical role of the nucleus in load sharing between disc components.

This study has separated the contribution of the AF and NP to disc behaviour and has evaluated the effect of extreme structural changes to the nucleus. This furthers understanding on the load-sharing in the disc. Altering the structure of the nucleus affects this relationship between spinal structures, subjecting them to increased loading and strain. These results offer an interpretation of how degenerative changes to the intervertebral disc such as depressurisation after herniation, or tissue stiffening from age-related degenerative factors affect disc behaviour.

## Supplemental Material

sj-docx-1-pih-10.1177_09544119241272915 – Supplemental material for The effect of structural changes on the low strain rate behaviour of the intervertebral disc Supplemental material, sj-docx-1-pih-10.1177_09544119241272915 for The effect of structural changes on the low strain rate behaviour of the intervertebral disc by Samantha Hayward, Patrick S Keogh, Anthony W Miles and Sabina Gheduzzi in Proceedings of the Institution of Mechanical Engineers, Part H: Journal of Engineering in Medicine

sj-jpg-2-pih-10.1177_09544119241272915 – Supplemental material for The effect of structural changes on the low strain rate behaviour of the intervertebral disc Supplemental material, sj-jpg-2-pih-10.1177_09544119241272915 for The effect of structural changes on the low strain rate behaviour of the intervertebral disc by Samantha Hayward, Patrick S Keogh, Anthony W Miles and Sabina Gheduzzi in Proceedings of the Institution of Mechanical Engineers, Part H: Journal of Engineering in Medicine
