# Supplementary material for: The effect of structural changes on the low strain rate behaviour of the intervertebral disc
Source: Proc Inst Mech Eng H. 2024 Aug 24;238(8-9):851–64. doi: 10.1177/09544119241272915 (PMC11459866; doi:10.1177/09544119241272915)
Supplement: sj-docx-1-pih-10.1177_09544119241272915 – Supplemental material for The effect of structural changes on the low strain rate behaviour of the intervertebral disc [file sj-docx-1-pih-10.1177_09544119241272915.docx]

Supplementary material

A set of four facial curettes and a single Fox dermal curette (round, 2mm) were used in this study to extract the nucleus material from the specimens through an annular incision. The curettes used are shown in Figure S1.
