# Supplementary figures and images for: The effect of structural changes on the low strain rate behaviour of the intervertebral disc
Source: Proc Inst Mech Eng H. 2024 Aug 24;238(8-9):851–64. doi: 10.1177/09544119241272915 (PMC11459866; doi:10.1177/09544119241272915)

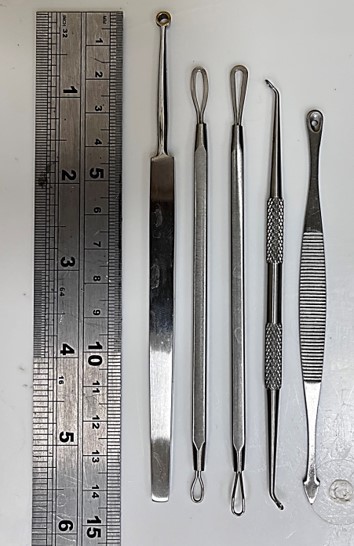

Supplement: sj-jpg-2-pih-10.1177_09544119241272915 – Supplemental material for The effect of structural changes on the low strain rate behaviour of the intervertebral disc [file sj-jpg-2-pih-10.1177_09544119241272915.jpg]
